# Pattern of β-Diversity and Plant Species Richness along Vertical Gradient in Northwest Himalaya, India

**DOI:** 10.3390/biology11071064

**Published:** 2022-07-18

**Authors:** Zishan Ahmad Wani, Sajid Khan, Jahangeer A. Bhat, Akhtar H. Malik, Tahira Alyas, Shreekar Pant, Sazada Siddiqui, Mahmoud Moustafa, Ahmad Ezzat Ahmad

**Affiliations:** 1 Conservation Ecology Lab, Department of Botany, Baba Ghulam Shah Badshah University, Rajouri 185234, Jammu and Kashmir, India; zishanwani786@gmail.com; 2 Conservation and Molecular Biology Lab, Department of Botany, Baba Ghulam Shah Badshah University, Rajouri 185234, Jammu and Kashmir, India; sajidkhan717@gmail.com; 3 College of Horticulture & Forestry, Rani Lakshmi Bai Central Agricultural University, Jhansi 284003, Uttar Pradesh, India; jahan191@gmail.com; 4 Centre for Biodiversity and Taxonomy, University of Kashmir, Srinagar 190006, Jammu and Kashmir, India; ecoakhtar@gmail.com; 5 Department of Botany, Lahore College for Women University Lahore, Punjab 54000, Pakistan; tahirailyas258@gmail.com; 6 Centre for Biodiversity Studies, Baba Ghulam Shah Badshah University, Rajouri 185234, Jammu and Kashmir, India; 7 Department of Biology, College of Science, King Khalid University, Abha 61413, Saudi Arabia; mfmostfa@kku.edu.sa (M.M.); aabdelrahman@kku.edu.sa (A.E.A.); 8 Department of Botany and Microbiology, Faculty of Science, South Valley University, Qena 83523, Egypt; 9 Department of Theriogenology, Faculty of Veterinary Medicine, South Valley University, Qena 83523, Egypt

**Keywords:** Himalayan region, elevation gradient, species richness, β-diversity, Renyi diversity profile

## Abstract

**Simple Summary:**

Elevation has a significant impact on the distribution of plant species. However, the structure and distribution patterns of Himalayan vegetation are poorly explored, and research on species composition along an elevation gradient in these mountain ranges is still deficient. Plant species richness is supposed to diminish with altitude, although numerous scientists have found a peak in species richness at mid-elevation, yielding a humped relationship. Many studies along the Himalayan altitudinal gradients have been conducted in order to better understand large-scale biogeographical patterns as well as what drives them, but no clear pattern has emerged. In order to understand how elevation affects plant species, we focused on species diversity, species composition and β-diversity, which allow for the interpreting of different patterns along the elevations. It was found that all these components of diversity vary significantly with the change in altitude.

**Abstract:**

The structure and distribution patterns of Himalayan vegetation are poorly explored, and research on species composition along the elevation gradient in these mountain ranges is still deficient. The current study was undertaken to analyze the variation and pattern of plant species composition along a vertical gradient in northwestern Himalaya, India. A total of 18 sites were selected along an elevation gradient ranging from 2200 to 3900 m asl positioned at an interval of 100 m. The Renyi diversity profile, non-metric multidimensional scaling based on the Bray–Curtis dissimilarity metric and beta diversity components among the elevation belts were calculated. Furthermore, to study the influence of altitude on species richness and diversity, a generalized additive model was created. Two hundred and ten plant species representing 66 families and 147 genera were recorded. The Renyi diversity profiles show that the lower and mid-altitudes had rich species diversity. The results of the non-metric multidimensional scaling analysis show a considerable variation in the total plant species composition among the studied elevation belts. The observed multiple-site Sorensen dissimilarity index across the studied elevation belts was very high. The contribution of species replacement or the turnover component to the observed dissimilarity was much higher than the nestedness component. Furthermore, the herbaceous and tree richness showed a significant decrease with increase in elevation; however, the richness of shrubs showed a bimodal pattern. The present study increases our understanding of the trends and patterns of species richness along the vertical gradient in the Himalayan region.

## 1. Introduction

Mountainous ecosystems, because of their rich floristic diversity, have always fascinated ecologists. Mountainous ecosystems provide steep elevational gradients, and several studies have been carried out to describe the patterns of species richness along the vertical gradients [1,2]. Some workers have reported a monotonous decrease in species richness along the vertical gradients [3,4,5,6,7,8] and some have reported hump-shaped patterns [9,10,11,12]; thus, no definite pattern has been observed. The Himalayas are one of the most diverse and expansive mountain ecosystems in the world [13] and one of the most important biodiversity hotspots. The Himalayan mountain range represents the highest elevational gradient and is bestowed with exceptionally rich and distinctive biodiversity [14]. The spatial variation in species diversity and its underlying mechanisms are a fundamental but still unsolved mystery in ecology [15] and mountainous regions such as the Himalayas are ideal for such studies. The most significant ecological features of forest ecosystems are species richness and composition, which display fluctuations in response to environmental conditions [16,17,18,19]. The composition, structure and distribution patterns of plant species within a community in mountainous regions are determined by a combination of variables including vegetation types, aspects and elevation [20,21,22]. Elevation is among the most significant factors having a robust impact on the vegetation structure in mountain ecosystems such as the Himalayas [22,23]. Elevation is a complex collection of related meteorological variables that are strongly linked to a variety of other environmental properties such as soil profile, nutrients and moisture [24]. Furthermore, within a given altitude, co-factors such as topography, aspect, slope and edaphic parameters have an impact on forest composition [25]. As a result, changes in altitude result in the formation of distinct microclimatic conditions, which promote diversity in species composition [26,27,28].

Plants have long served as a significant object for studying the elevational richness pattern and its underlying mechanism, owing to their distribution and ease of surveying [15]. The up-hill migration of plant species has been predicted and demonstrated by numerous workers in response to changing climatic circumstances, and hence investigating the patterns of species richness and composition is a global concern [29,30]. In the Himalayan mountain ecosystems, climatic zones may change rapidly and this is reflected by prominent changes in vegetation structure even at small distances [6]. Although many studies along the Himalayan altitudinal gradients have been conducted in order to better understand large-scale biogeographical patterns along with what drives them, [31,32,33,34,35,36,37,38,39,40,41,42,43,44] no clear pattern has emerged, as most of these studies attempted to document the pattern of species and composition at lower or higher elevational zones. Therefore, the floristic characteristics and plant diversity patterns in the Himalayan region in general and the Indian Himalayan region in particular are still data-deficient and thus poorly understood [22,45]. Furthermore, the Pir Panjal range, a remote area of the Indian Himalayan region, has remained unexplored owing to its remoteness, inaccessibility and difficult terrains. Therefore, the present study aimed to study the variation and patterns of plant species composition along a broad vertical gradient (2200–3900m) in the Gulmarg Wildlife Sanctuary, a part of the Pir Panjal range of the Northwestern Indian Himalayan Region, with the following objectives;

(a) assess the floristic diversity within the study area;
(b) calculate the total species diversity at each elevation belt;
(c) explore the deviation in plant species composition among the elevation belts;
(d) calculate the beta diversity as well as its underlying components among the elevation belts;
(e) and study the influence of elevation on species richness and diversity.

## 2. Materials and Methods

### 2.1. Study Area

**Figure 1:**
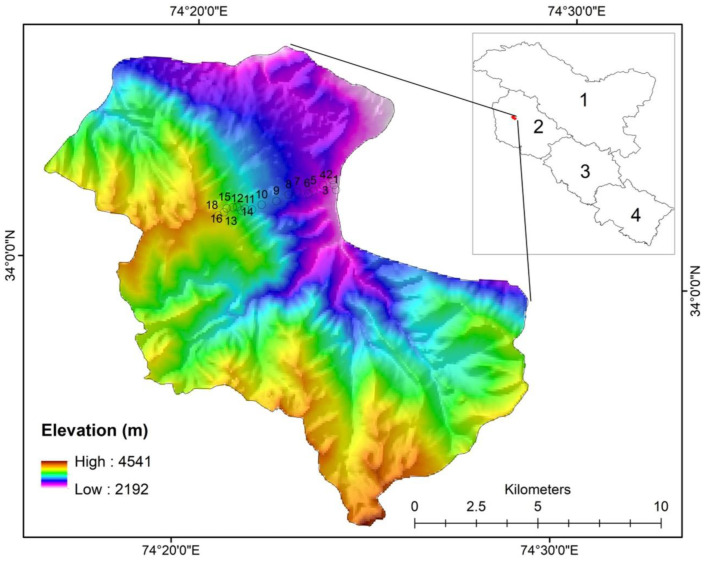
Location of study area showing sampling sites.

The Gulmarg Wildlife Sanctuary (GWLS) falls 25 Kms to the southwest of the Baramulla district of Jammu and Kashmir (74°17′ to 74°79′ N latitude and 34°55′ to 34°60′ E longitude) with a wide altitudinal range (Figure 1). It is surrounded by the Jhelum Valley Forest Division, Baramulla, to the north; the Forest Division of Poonch to the south; the Drang and Badrakoot forests of the Special Forest Division, Tangmarg, to the east; and the Special Forest Division of Tangmarg and Baba Reshi to the West. The study area has a continental temperate climate with an annual average rainfall of 1049 mm yr-1. July is the hottest month of the year, with temperatures averaging 20 degrees Celsius. The coldest month is January, with temperatures as low as −6 °C (http://www.imd.gov.in/pages/main.php, accessed on 13 June 2021). Lower altitudes (Tangmarg) have temperate climates; the mid-altitudes (Gulmarg-Khilenmarg) exhibit a gradual climatic gradient from temperate and sub-alpine climates; and in the higher altitudes, snow remains in ridges and pockets late, from the end of June up to September.

### 2.2. Site Selection and Data Collection

Three transects along the altitude, i.e., Tangmarg to Gulmarg (2200–2500 m), Gulmarg to Khilenmarg (2501–3100 m) and Khilenmarg to the Afarwat top (3101–3900 m) covering the forest, sub-alpine and alpine zones of the sanctuary were selected. A total of 18 accessible sites having the least anthropogenic disturbances were selected along the transects positioned at an interval of 100 m. The composition of each site was evaluated by organized sampling using the quadrat method, as this method was more unbiased and convenient [40,46]. At each site, 10 (10 × 10 m), 10 (5 × 5 m) and 20 (1 × 1 m) nested quadrats were laid for trees, shrubs and herbs respectively. Thus, a total of 740 quadrats (360 for herbs, 180 for shrubs as well as trees) were laid. Furthermore, the plant species found during the sampling were collected, tagged and brought to the lab where each plant specimen was pressed, dried and mounted on a herbarium sheet. Later each plant species was identified up to the species level with the help of local floras [47,48,49] and further authentication of plant species was performed at the Centre for Biodiversity and Taxonomy, University of Kashmir. The nomenclature and family of each plant species were retrieved from POWO (https://powo.science.kew.org, assessed on 25 March 2021–23 October 2021) and WFO (http://worldfloraonline.org, assessed on 25 March 2021–23 October 2021), respectively.

### 2.3. Data Analysis

To calculate the total species diversity at each elevation belt, the Renyi diversity profile technique was used using the vegan 2.5–7 package [50]. The Renyi diversity profile is generally a function-dependent parametric category of diversity indices that allows for visual comparison of the graphical ordering of communities under investigation [50,51,52]. We calculated the Renyi diversity profile values (Hα) from the average abundance values of the ten quadrats laid at each elevation belt and a scaling parameter (α) that ranged from zero to infinity [53] using the formula;

$$H_{\alpha }=1\;\frac{1}{1-\alpha }\ln\sum_{i=1}^{S}pi^{\alpha }$$

where pi represents the average abundance of each species from the ten quadrats and α is the scaling parameter [50]. The values of the Renyi profile at the scales of 0, 1, 2 and infinity (∞) generally refer to species richness (S), Shannon index (H’), Simpson index and Berger-Parker index, respectively [51,52]. In the present study, the diversity values were calculated for the default α scale of 0, 0.25, 0.5, 1, 2, 4, 8, 16, 32, 64 and infinity (∞) and we plotted the corresponding Renyi diversity profiles for each of the studied elevation belts. According to the Renyi diversity profile, a community is generally considered more diverse if its values over the entire range of profiles are higher than other communities [50,51,52].

To explore the deviation in the total plant species composition between the studied elevation belts, non-metric multidimensional scaling (NMDS) based on the Bray–Curtis dissimilarity metric was performed using the vegan 2.5–7 package in R Software [50]. This ordination technique transforms information from n-dimensional data (e.g., multiple species’ abundances across a number of sites) into a few components for the sake of easy visualization and interpretation [54]. Additionally, the associated stress value for the NMDS was calculated to evaluate its accuracy. The stress value varies between 0 (no stress) and 1 (complete lack of fit) with values greater than 0.3 generally considered as unsatisfactory [53,54,55]. The associated NMDS plots were generated, wherein each ellipse or polygon (along with its associated centroid) corresponded to a given elevation belt and the observed distance between any two centroids represented the degree of dissimilarity in species composition between the corresponding elevation belts. Moreover, the overall size of each ellipse was proportional to the degree of dissimilarity quadrats within a particular elevation belt [55]. Additionally, the degree of compositional differences between the studied elevation belts was quantified using the permutational analysis of variance (PERMANOVA). Using PERMANOVA, both abundance-based Bray–Curtis and incidence-based Jaccard indices were used to study whether the observed patterns in the compositional dissimilarity were due to species relative abundances or instead due to the presence and/or absence of species [55]. We calculated the associated statistical significance by setting the alpha at 0.05 and calculating 999 permutations.

To calculate the overall and the pairwise beta diversity components between the elevation belts as well as their underlying components (turnover and nestedness), we adopted the betapart 1.5.4 package [56] using the presence–absence data. The method we used segregates the observed dissimilarity (βSOR) into two additive components, namely species turnover or replacement (βSIM), which accounts for the replacement of one species in a site by another species in the other site, and nestedness (βSNE), which accounts for the loss or gain of species between the two sites under investigation [57]. The clustering of elevation belts based on the β_SIM_ and β_SNE_ components of the total plant species dissimilarity among the studied elevation belts was also carried out by using the betapart 1.5.4 package.

Furthermore, to study the influence of elevation on species richness and species diversity (Shannon), we performed the generalized additive model using the ‘mgcv’ package in R [58]. The species richness at each elevation belt was computed as the total number of species present at any location in that elevation belt, whereas the Shannon diversity was calculated based on the average abundance data from the ten quadrats that were laid at each elevation belt. The generalized additive model analysis was performed separately for the total, herbaceous, shrubs and trees.

## 3. Results

### 3.1. Floristic Diversity

**Figure 2:**
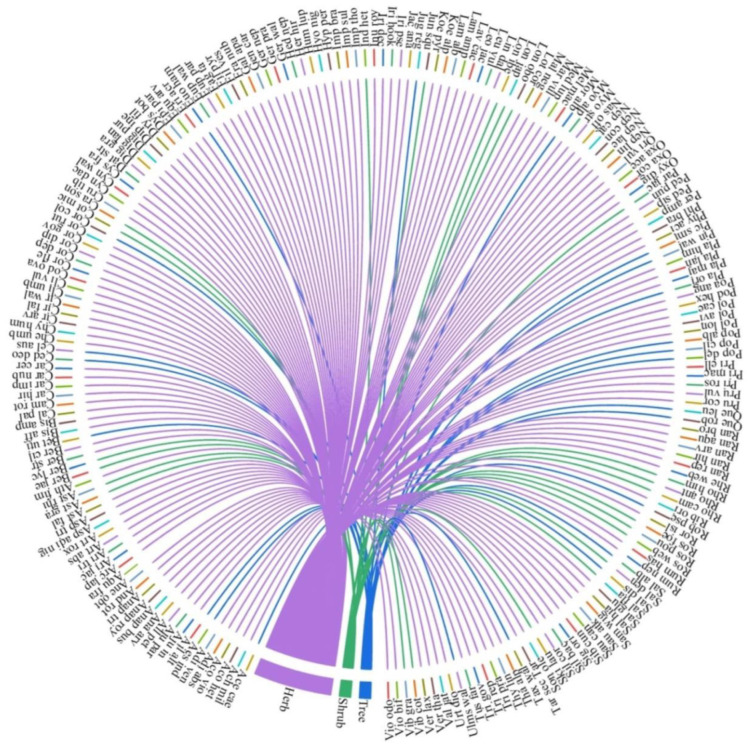
Growth form of the documented plant species.

During the present study, 210 plant species representing 66 families and 147 genera were identified from the study area (Table S1). Asteraceae, Lamiaceae and Ranunculaceae were the dominant families with 28, 17 and 11 species respectively (Table S2). *Ranunculus* was the dominant genus with five species followed by *Nepeta* with five species. Genera *Anaphalis*, *Circium*, *Corydalis*, *Digitalis*, *Geranium*, *Impatiens*, *Iris*, *Lonicera*, and *Plantago* were represented by three species each. Life-forms were dominated by the herbaceous forms (including ferns) (79.04%), followed by shrubs (9.52%) and trees (11.4%) (Figure 2).

### 3.2. Species Diversity at Each Elevation Belt

**Figure 3:**
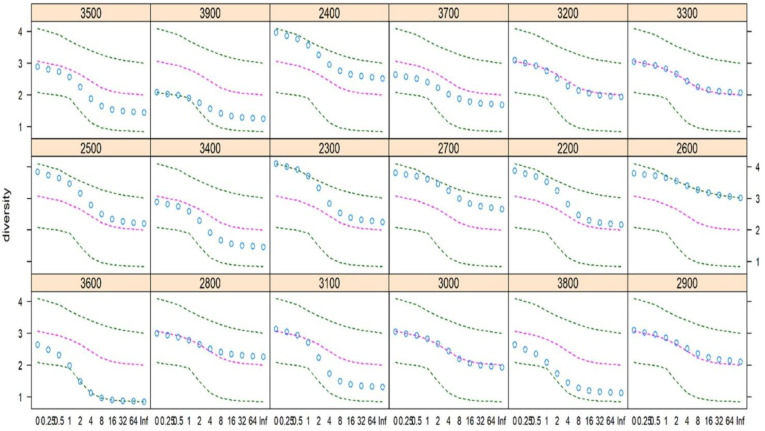
Renyi diversity profiles of total plant species in the studied elevation belts. The dots represent the diversity value for each elevation belt; the outer two dashed lines, the extremes; and the inner pink line, the median in the data.

The Renyi diversity profiles for the studied elevation belts are presented in Figure 3. As revealed from the diversity profiles, higher plant species diversity was observed at 2300 m at the scaling parameter (α) values between 0 and 1, whereas the diversity was higher at an elevation belt of 2600 m for the rest of the scaling parameter (α) values. Contrary to this, the lowest species diversity was observed at an elevation belt of 3900 m for the scaling parameter (α) values between 0 and 1, but at the 3600 m elevation belt for the rest of the observed scaling parameter (α) values (Figure 3). In this way, our way of calculating the species diversity was unique and more robust than arbitrarily chosen single-diversity indices, which usually result in biased generalizations.

### 3.3. Deviation in Species Composition among Elevation Belts

**Figure 4:**
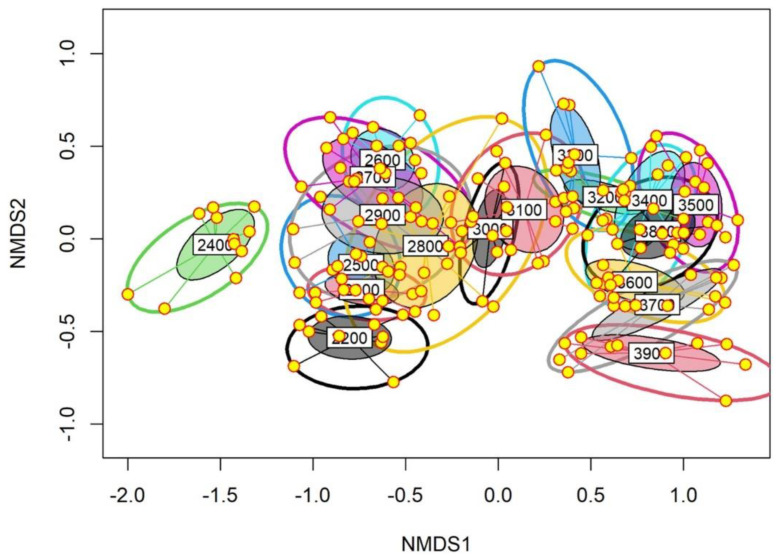
Non-metric dimensional scaling (NMDS) plot showing differences in total plant species composition between the studied 100 m elevation belts. For each ellipse, the size is proportional to within-group dissimilarity or variability, while the degree of overlap between any two ellipses shows the associated community similarity between their respective vegetation.

The results of NMDS analysis revealed a significant extent of dissimilarity in the total plant species composition between the studied elevation belts. More specifically, a varying degree of overlap was observed between the lower elevation belts (2300–2900 m) and also between the higher elevation belts (3200–3900 m) (Figure 4). An overlap between the two mid-elevational belts (3000 and 3100 m) forming a separate cluster in between the lower and higher elevation belts was observed. Furthermore, the 2400 m elevation belt was different from all other elevation belts, forming a non-overlapping eclipse. However, the species composition of the extreme lower and higher elevation belts differed significantly as reflected by the non-overlapping ellipses (Figure 4). Moreover, the associated stress level for the NMDS plot was very low (0.12) for the first three dimensions. Furthermore, the PERMANOVA analysis indicated that the observed differences in the species for studied elevation belts were significantly different from each other, as evident from both the Bray–Curtis (F = 30.75; *p* < 0.01) and the Jaccard (F = 17.99, *p* < 0.01) indices, which in turn indicated that the observed differences in species composition across the studied elevation belts were driven by both changes in the species number and the associated relative abundances.

### 3.4. Spatial β-Diversity Patterns among Elevation Belts

**Figure 5:**
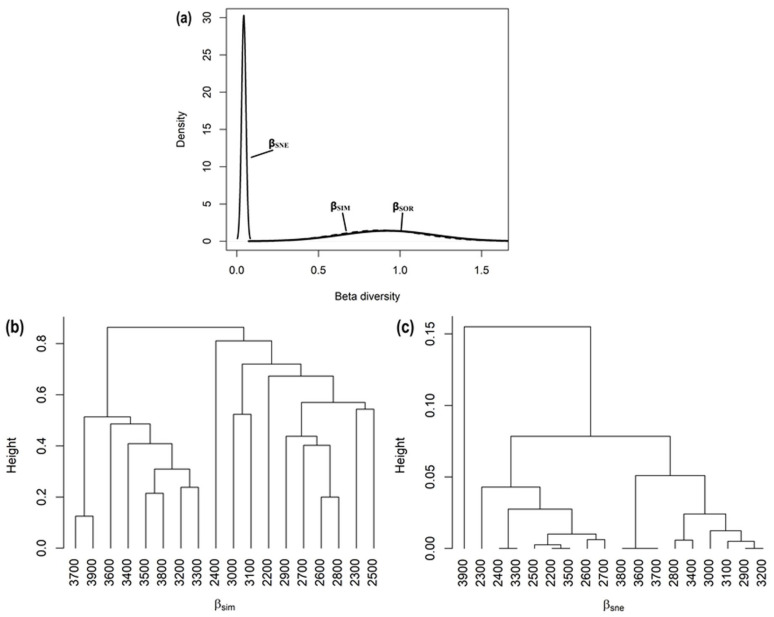
Multiple-site dissimilarities across the studied elevation belts using total plant species composition. (**a**) Partitioning of total dissimilarity (β_SOR_: black solid line) into species turnover or replacement (β_SIM_: dashed line) and nestedness (β_SNE_: grey solid line) components; (**b**) average clustering based on β_SIM_ components of total plant species dissimilarity among the studied elevation belts; and (**c**) β_SNE_ components of total plant species dissimilarity among the studied elevation belts.

Overall, the observed multiple-site Sorensen dissimilarity index across the studied 100 m elevation belts was very high (β_SOR_ = 0.92). The contribution of species replacement or the turnover component to the observed dissimilarity was much higher (β_SIM_ = 0.88) when compared with the nestedness component (β_SNE_ = 0.04). These results in turn suggest that the observed dissimilarity was more likely due to species replacement (turnover) than species richness difference (nestedness) (Figure 5a). Furthermore, a considerable variation was observed for the pair-wise Sorensen dissimilarity index. Once again, this resulting pair-wise dissimilarity was more like a consequence of species replacement (β_SIM_) compared to the nestedness (β_SNE_) component in a majority of the comparisons. Furthermore, the cluster analysis based on the turnover or replacement component of dissimilarity (β_SIM_) indicated the 2400 m elevation belt to be highly dissimilar from the rest of the studied elevation belts in terms of total plant species composition, followed by the 3000 and 3100 m elevation belts (Figure 5b). Contrary to this, cluster analysis approach based on the nestedness component of dissimilarity (β_SNE_) revealed the composition at the 3900 m elevation belt to be highly dissimilar from the rest of the studied elevation belts (Figure 5c).

### 3.5. Pattern of Species Richness and Diversity along Elevation Gradient

**Figure 6:**
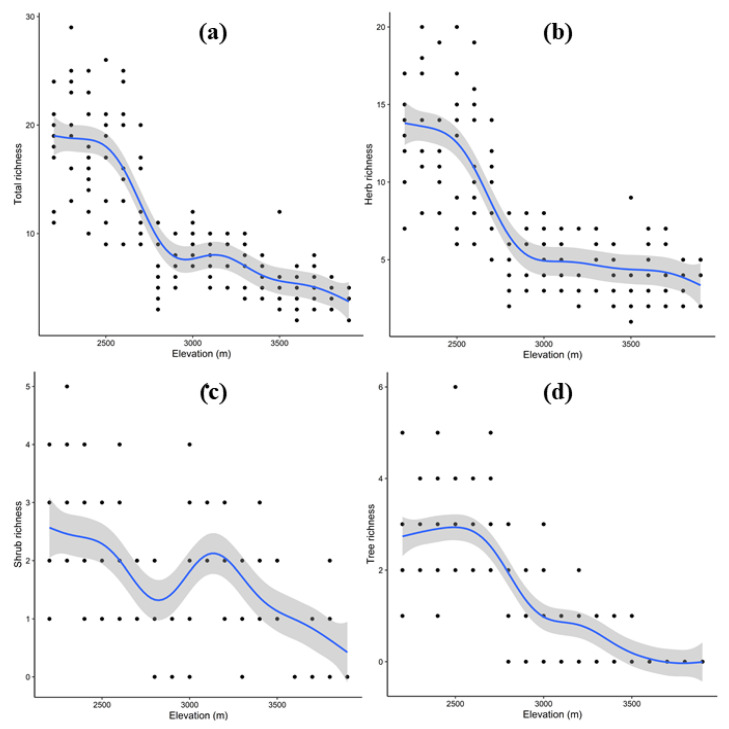
Relationship between elevation and plant richness patterns for total (**a**), herb (**b**), shrub (**c**) and tree (**d**) recorded at 100 m elevation belts. Shown are the best-fitted regression splines from generalized additive model (solid blue line), standard errors (grey shadings) and observations (black dots).

The total plant richness showed a significant decrease with increase in elevation (R^2^ = 0.74, *p* value < 0.001), being higher at lower elevations and then decreasing abruptly until 2900 m, whereupon it followed a flat plateau up to 3300 m and then decreased again (Figure 6a). Similarly, the herbaceous and tree richness showed a significant decrease with increase in elevation (R^2^ = 0.68, *p* value < 0.001), being higher at lower elevations and then decreasing consistently (Figure 6b–d). However, the shrub richness showed a bimodal pattern (R^2^ = 0.32, *p* value < 0.001), peaking at < 2500 m and at 3000–3200 m (Figure 6c).

**Figure 7:**
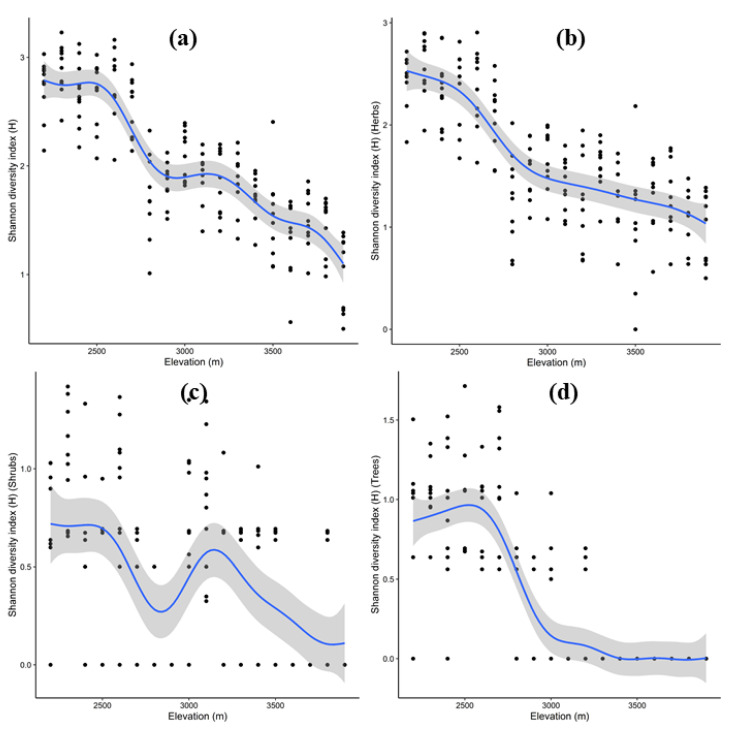
Relationship between elevation and Shannon diversity index for total (**a**), herb (**b**), shrub (**c**) and tree (**d**) recorded at 100 m elevation belts. Shown are the best-fitted regression splines from generalized additive model (solid blue line), standard errors (grey shadings) and observations (black dots).

The Shannon diversity index for total plant species varied significantly with elevation (R^2^ = 0.74, *p* value < 0.001). More specifically, a bimodal pattern was observed, peaking between 2200 and 2500 m and again at 3000–3200 m (Figure 7a). Similarly, the Shannon diversity index for herbs varied significantly with elevation (R^2^ = 0.63, *p* value < 0.001), being higher at lower elevations (2200–2400 m) and then decreasing abruptly until 2900 m, after which a more or less flat plateau formed (Figure 7b). Once again, the Shannon diversity index for shrubs varied significantly with elevation (R^2^ = 0.26, *p* value < 0.001), forming a bi-modal pattern, peaking between 2200 and2500 m and again at 3000–3200 m (Figure 7c). Finally, the Shannon diversity index for tree species decreased significantly with elevation (R^2^ = 0.67, *p* value < 0.001), generally being higher at lower elevations between 2400 and 2600 m (Figure 7d).

## 4. Discussion

The study area was well represented, with 210 plant species (20 trees, 24 shrubs and 166 herbs), which was higher than earlier reports by Shaheen et al. [17]; Dar and Sundarapandian [22]; and Bano et al. [38] from different areas of the Himalayan region. Furthermore, Asteraceae, Lamiaceae and Ranunculaceae were the most dominant families in terms of number of species and have also been reported as the dominant families in the Himalayan region by other workers [59,60,61,62]. The Renyi diversity profiles calculated during the present study show that maximum species richness and diversity were found at lower and mid-elevations in comparison to higher elevations, coinciding with the results of Sharma et al. [63], Bhat et al. [64] and Rawat et al. [65]. The present study revealed that trees and shrubs were represented up to a limited altitude of 3300 m asl, but cushion-forming shrubs such as *Juniperus squamata* and *Rhododendron anthopogon* were found up to elevation peaks. Furthermore, a dominance of herbaceous forms throughout the entire elevation gradient, in terms of species richness, was observed; in the Himalayan region, frost kills tree seedlings and acts as a limiting factor in the spread of arboreal species at higher elevations [66,67]. Herbs, on the other hand, are seen to be the most sophisticated and effective growth forms due to their capacity to adapt to a wide range of environmental conditions. Some herbaceous plant species can even withstand the whole range of environmental conditions at severe gradients. In the present study, plant species such as *Trifolium repens*, *Trifolium pratense*, *Circium arvense*, *Urtica dioica* and *Myosotis* sp., were found to have more diverse altitudinal ranges. *Circium arvense* was found at 17 sites from 2200 to 3800 m amsl. However, native and endemic plant species such as *Aconitum heterophyllum, Saussurea costus*, *Aconitum violaceum*, *Aconitum violaceum*, *Angelica glauca*, *Bistorta affinis*, *Dolomiaea macrocephala* and *Inula royleana* were more frequent at higher altitudes.

Variation in species composition with varying elevation is a common phenomenon in the Himalayas and other mountainous ecosystems [68,69,70,71]. A major proportion of the disparity in species composition between elevation belts is attributable to micro-environmental variations [72,73]. The present study revealed a significant difference between species compositions along the altitudinal gradient. The composition of plant species at different elevation belts in the present study area (Table 1) was most likely influenced by alterations in temperature, rainfall, wind, solar radiation and humidity with changing altitudes. These variables led to robust similarity of environmental conditions and species composition within smaller elevation bands [74], whereas greater elevation differences (2200 & 3900 m in present study) increased environmental heterogeneity and resulted in additional species with different niche preferences [75]. Furthermore, the two elevation belts (3000 and 3100 m) forming the ecotone had species from both forest as well as alpine ecosystems, thus forming a separate cluster in between the lower and higher elevation belts. In addition, the species composition at the 2400 m elevation belt was significantly different from the rest of the elevation belts, which may be attributed to observed anthropogenic disturbances (overgrazing, deforestation, trampling, camping, animal and human wastes) at this elevation belt. Most of the species recorded at the 2400 m elevation belt were invasive alien species including *Leucanthemum vulgare*, *Rumex nepalensis*, *Digitalis glandiflora*, *Polygonum aviculare* and *Sambucus wightiana*. Because of anthropogenic disturbances, invasive alien species had the opportunity to expand in their habitat ranges [30] and to gradually displace slower-growing native species that need more stable environments. As a result, most alien species, which are often restricted to lower elevations, are expected to move up to higher elevations under a scenario of increasing human disturbances and climate change [30,76].

The present study shows that the elevation influences the β-diversity considerably along an elevation gradient. We also found that the β_SIM_ was more significant for β-diversity than the β_SNE_. Furthermore, the β-diversity pattern shows that the species turnover rate was lower at mid-altitudes in comparison to the lower and higher altitudes, which is in coherence with Rawal et al. [77]. The mid-altitudinal range had the most species that shared habitats with other elevation ranges and the extreme elevational ranges shared fewer species with lower ranges, thus having a high species replacement rate. The minimum species turnover rate at lower and mid-elevational gradients explained that higher altitudes have different habitats and environmental conditions enabling the differential establishment of unique plant species. Ecological restrictions limiting species occurrences and evolutionary restraints allied with adaptations to site-specific ecological factors could both contribute to community structuring [78]. The species composition at high altitudes was significantly distinctive compared to lower altitudes (Table 1). The variation in β_SIM_ was less within lower or higher elevation belts, while this variation was greater between lower and higher elevation belts. As elevation increased, the species replacement rate between sampling sites tended to increase, reaching >90% at the highest elevations (2200–3900). A reduced rate of species replacement across sites at mid-elevations shows that environmental circumstances were more conducive to the coexistence of more species [79]. The high rate of species replacement across extreme sampling sites, on the other hand, indicated of significant environmental changes as well as adaptations between functional categories [80]. The most homogeneous species pool was within the higher elevation belts, possibly due to site-specific environmental conditions [81,82,83,84]. Higher elevations had a more or less monotonic or homogeneous composition dominated by one or few species due to site-specific environmental conditions favorable only for a few adaptable species [83,84]. During the present study, *Bistorta affinis*, *Juniperus squamata* and *Rhododendron anthopogon* were found to be most common species at higher altitudes.

Elevational transects have long been hailed as good models of larger-scale biological trends because they compress significant climate changes into relatively short distances [85]. Vegetation in mountainous areas varies in physiognomy, species richness and diversity along altitudinal gradients [86,87,88,89]. In the present study, total species richness and diversity followed a unimodal pattern—decreasing with an increase in elevation gradient, which is considered a general pattern [90]. Gomez-Diaz [91] and Rawat et al. [7] also recorded similar trends in central Veracruz, Mexico and the western Himalayas, respectively. However, we found that shrubs peaked at mid-elevations, which may be due to extra space at these elevations due to sparse tree canopy. At higher altitudes, only a few patches of shrubs and alpine rangelands were found that lacked the environmental conditions favoring high species diversity. Meager species richness and diversity at higher elevations may be due to the harsh environmental conditions (extreme cold and glaciated soils in the present study) restricting plant growth, and subsequently, loss of diversity [92,93]. Thus, at higher altitudes, the plant species were exposed to various eco-physiological restraints such as high snowfall, short growing season, low temperature, low ecosystem productivity and loss of habitat diversity, which in turn reduced the species richness and diversity [94,95]. In contrast, the greater species richness and diversity at lower and mid-elevations was largely correlated with moisture availability, optimum temperature and favorable environmental conditions [96]. Furthermore, the increase and reduction in species richness at lower and higher elevations, respectively, was governed by increased productivity at lower and mid-elevations compared to that at higher elevations [44]. Additionally, at higher elevations, organic matter decays slowly in low-temperature soils, resulting in an oversupply of humus, which limits soil production and lowers vascular plant nutrient absorption [97,98]. In comparison to low-altitude plant species, alpine plant species employ distinct tactics for surviving in a hostile environment. Increased resource investment in reproductive and belowground sections, increased display area to attract pollinators and improved dispersal systems are among these techniques [99].

## 5. Conclusions

In order to understand how elevation affects plant species, we focused on species diversity, species composition and β-diversity that allowed for interpreting different patterns along the elevations. We found that all these components of diversity varied significantly with the change in altitude. The species diversity at lower and mid-elevation belts was higher than the upper elevation belts with considerable variation in species composition at the extreme lower (2200 m asl) and extreme higher (3900 m asl) elevation belts. Furthermore, it is obvious from the present study that the change in elevation considerably influences the β-diversity among the elevation belts and that species replacement is more significant for β-diversity than species nestedness. The herbaceous and tree richness and Shannon diversity index show a significant decrease with increase in elevation whereas the shrub richness and Shannon diversity index for shrubs show a bimodal pattern. As the structure and distribution patterns of Himalayan vegetation are poorly explored, and research on species composition along an elevational gradient in these mountain ranges is still deficient, the present study increases our understanding of the trends and patterns of species richness along the vertical gradient in the Himalayan region.

## Figures and Tables

**Table 1:** Dominant plant species along the elevation gradient in GWLS, Kashmir Himalaya.

Altitude (m)	Most Frequent Species
2200	*Pinus wallichiana*; *Capsella bursa-pastoris*; *Geum roylei*; *Leucanthemum vulgare*
2300	*Abies pindrow*; *Virburnum grandiflorum*; *Lamium album; Valeriana jatamansii*
2400	*Abies pindrow*; *Leucanthemum vulgare*; *Rumex nepalensis*; *Polyygonum aviculare*
2500	*Aesculus indica; Parrotiopsis jacquemontiana, Prunus cornuta*
2600	*Abies pindrow*; *Bistorta amplexicaulis*; *Skimmia anquetilia*; *Arisaema jacquemontii*
2700	*Abies pindrow*; *Polygonum aviculare*; *Rumex nepalensis; Epimedium elatum*
2800	*Bistorta amplexicaulis*; *Cirsium arvensis*; *Cirsium falconeri*
2900	*Cirsium arvensis*; *Euphorbia wallichi*
3000	*Bupleurum candollei*; *Iris hookeriana*; *Cirsium falconeri*; *Thymus linearis*
3100	*Abies pindrow*; *Rhododendron campanulatum*; *Salix denticulata*; *Euphorbia wallichii*; *Bistorta amplexicaulis*
3200	*Betula utilis*; *Salix denticulata*; *Aconitum heterophyllum*; *Bupleurum candollei*
3300	*Betula utilis*; *Bistorta amplexicaulis*; *Iris hookeriana*
3400	*Sibbaldia cuneata*; *Saussurea atkinsonii*; *Bergenia ciliata*
3500	*Juniperus squamata*; *Betula utilis*; *Salix denticulata*; *Salvia hians*; *Swertia petiolata*; *Phlomis bracteosa*
3600	*Juniperus squamata*; *Rhododendron anthopogon*; *Anaphalis roylei*; *Cordiofontis flexuosa*; *Thymus linearis*
3700	*Bisorta affinis*; *Taraxacum sect Taraxacum*; *Viola biflora*
3800	*Juniperus squamata*; *Rhododendron anthopogon*; *Bistorta affinis; Rheum webbianum*
3900	*Bistorta affinis*; *Bergenia ciliata*

## Data Availability

Not applicable.

## Supplementary Materials

The following supporting information can be downloaded at: https://www.mdpi.com/article/10.3390/biology11071064/s1, Table S1: List of the documented plant species from the Gulmarg Wildlife Sanctuary, Northwest Himalaya; Table S2: Family wise contribution of the documented plant species.
