# Supplementary material for: Pattern of β-Diversity and Plant Species Richness along Vertical Gradient in Northwest Himalaya, India
Source: Biology (Basel). 2022 Jul 18;11(7):1064. doi: 10.3390/biology11071064 (PMC9312975; doi:10.3390/biology11071064)
Supplement: Supplementary file 1 [file biology-11-01064-s001.zip › Suppl S1.pdf]

Suppl. S1

Table S1. List of the documented plant species from the Gulmarg Wildlife Sanctuary, Northwest Himalaya

Taxa	Family	Habit
Acer caesium Wall. ex Brandis	Sapindaceae	Tree
Achillea millefolium L.	Compositae	Herb
Achnatherum sibiricum (L.) Keng ex Tzve.	Poaceae	Herb
Aconitum heterophyllum Wall. ex Royle	Ranunculaceae	Herb
Aconitum violaceum Jacq. ex Stapf.	Ranunculaceae	Herb
Adiantum abscissum Schrad.	Pteridaceae	Herb
Adiantum venustum D. Don	Pteridaceae	Herb
Aesculus indica (Wall. ex Camb.) Hook.	Sapindaceae	Tree
Ailanthus altissima (Mill.) Swingle	Simaroubaceae	Tree
Ajuga integrefolia Buch.-Ham. ex D. Don	Lamiaceae	Herb
Ajuga parviflora Benth.	Lamiaceae	Herb
Alliaria petiolata (M. Bieb.) Cav. & Grande	Brassicaceae	Herb
Anagallis arvensis (M. Bieb.) Cavara & Grand	Primulaceae	Herb
Anaphalis busua Buch.-Ham. ex DC.	Compositae	Herb
Anaphalis roylei DC.	Compositae	Herb
Anaphalis triplinervis (Sims) C.B. Clarke	Compositae	Herb
Androsace rotundifolia Hardw.	Primulaceae	Herb
Anemonastrum obtusilobum (D. Don) Mosy.<br>Syn. *Anemone obtusiloba* D. Don	Ranunculaceae	Herb
Aquilegia fragrans Benth.	Ranunculaceae	Herb
Arctium lappa L.	Compositae	Herb
Arisaema jacquemontii Blume	Araceae	Herb
Arisaema propinquum (L.) Schott	Araceae	Herb
Artemisia absinthium L.	Compositae	Herb
Artemisia roxburghiana Besser	Compositae	Herb
Asplenium adiantum nigrum L.	Aspleniaceae	Herb
Asplenium trichomanes L	Aspleniaceae	Herb
Aster falconeri (C.B. Clarke) Hutch.	Compositae	Herb
Astragalus grahamianus Benth.	Fabaceae	Shrub
Astragalus rhizanthus Benth.	Fabaceae	Shrub
Athyrium fimbriatum T. Moore	Athyriaceae	Herb
Berberis jaeschkeana C.K. Sch.	Berberidaceae	Shrub
Berberis lycium Royle	Berberidaceae	Shrub
Bergenia ciliata (Haw.) Sternb.	Saxifragaceae	Herb
Bergenia stracheyi (Hook.f. & Thomp.) Engl.	Saxifragaceae	Herb
Betula utilis D. Don	Betulaceae	Tree
Bisorta affinis (D. Don) Greene<br>Syn. *Polygonum affine* D. Don	Polygonaceae	Herb
Bistorta amplexicaulis (D. Don) Greene<br>Syn. *Polygonum amplexicaule* D. Don	Polygonaceae	Herb
Bupleurum candollei Wall. ex DC.	Apiaceae	Herb
Caltha palustris L.	Ranunculaceae	Herb
Campanula rotundifolia L.	Campanulaceae	Herb
Cardamine hirsuta L.	Brassicaceae	Herb

Cardamine impatiens L.	Brassicaceae	Herb
Carex nubigena D. Don	Cyperaceae	Herb
Carpesium cernuum L.	Compositae	Herb
Cedrus deodara (Roxb. ex D. Don) G. Don	Pinaceae	Tree
Celtis australis L.	Cannabaceae	Tree
Chenopodium umbossum L.	Amaranthaceae	Herb
Chyrojasminum humile (L.) Banfi<br>Syn. *Jasminum humile* L.	Oleaceae	Shrub
Cirsium arvense (L.) Scop.	Compositae	Herb
Cirsium falconeri (Hook.f.) Petr.	Compositae	Herb
Cirsium wallichi DC.	Compositae	Herb
Clinopodium umbrosum (M.Bieb.) K. Koch	Lamiaceae	Herb
Clinopodium vulgaris L.	Lamiaceae	Herb
Codonopsis ovata Benth.	Campanulaceae	Herb
Cordiofontis flexuosa (Royle ex. Lindl.) G.L. Nesom<br>Syn. *Aster thomsonii* C.B. Clarke	Compositae	Herb
Cortia depressa (D. Don) C. Norman	Apiaceae	Herb
Corydalis diphylla Wall.	Papaveraceae	Herb
Corydalis govaniana Wall.	Papaveraceae	Herb
Corydalis rutifolia (Sm.) DC.	Papaveraceae	Herb
Corylus colurna L.	Betulaceae	Tree
Cotoneaster microphylla Wall. ex Lindl.	Rosaceae	Shrub
Crataegus songarica K. Koch	Rosaceae	Shrub
Crepis pulchra L.	Compositae	Herb
Crucihimalaya tibetica (Hook.f. & Thomp.) Sheh. Germ. & K. Koch<br>Syn. *Arabis tibetica* Hook.f. & Thomp.)	Brassicaceae	Herb
Cynodon dactylon (L.) Pers.	Poaceae	Herb
Cynoglossum wallichi G. Don	Boraginaceae	Herb
Cystopteris fragilis (L.) Bernh.	Cystopteridaceae	Herb
Datura stramonium L.	Solanaceae	Herb
Digitalis grandiflora Mill.	Plantaginaceae	Herb
Digitalis lanata Ehrh.	Plantaginaceae	Herb
Digitalis purpurea L.	Plantaginaceae	Herb
Dipsacus inermis Wall.	Caprifoliaceae	Herb
Dolomiaea costus (Falc.) Kasana & Panday<br>Syn. *Saussurea costus* (Falc.) Lipsch.	Compositae	Herb
Dryopteris filix-mas (L.) Schott	Dryopteridaceae	Herb
Dysphania botrys (L.) Mosy. & Clem.<br>Syn. *Chenopodium botrys* L.	Amaranthaceae	Herb
Epilobium *parviflorum* Schreb.	Onagraceae	Herb
Epimedium elatum C. Morren & Decne.	Berberidaceae	Herb
Equisetum arvense L.	Equisetaceae	Herb
Erigeron acris var. *multicaulis* (Wall. ex DC.) C.B. Clarke<br>Syn. *Erigeron multicaulis* Wall. ex DC.	Compositae	Herb
Euonymus hamiltonianus Wall.	Celestraceae	Tree
Euphorbia wallichii Hook.f.	Euphorbiaceae	Herb
Euphrasia parviflora Sch.	Orobanchaceae	Herb
Fagopyrum tataricum (L.) Gaertn.	Polygonaceae	Herb
Filago pyramidata L.	Compositae	Herb
Filipendula vestita (Wall. ex G. Don) Maxim.	Rosaceae	Herb
Fragaria nubicola (Lindl. Ex Hook.f.)	Rosaceae	Herb

Lac.		
Galium aparine L.	Rubiaceae	Herb
Gentiana carinata (D. Don ex G. Don) Griseb.	Gentianaceae	Herb
Geranium nepalense Sweet	Geraniaceae	Herb
Geranium pratense L.	Geraniaceae	Herb
Geranium wallichianum D. Don ex Sweet	Geraniaceae	Herb
Hedera nepalensis K. Koch	Araliaceae	Herb
Herniaria hirsuta L.	Caryohyllaceae	Herb
Humulus lupulus L.	Cannabaceae	Herb
Hyoscyamus niger L.	Solanaceae	Herb
Hypericum perforatum L.	Hypericaceae	Herb
Impatiens brachycentra Kar & Kir	Balsaminaceae	Herb
Impatiens sulcata Wall.	Balsaminaceae	Herb
Impatiens thomsonii Hook.f.	Balsaminaceae	Herb
Indigofera heterantha Wall. ex Brandis	Fabaceae	Shrub
Inula royleana DC.	Compositae	Herb
Iris decora Wall.	Iridaceae	Herb
Iris hookeriana Foster	Iridaceae	Herb
Iris pseudacorus L.	Iridaceae	Herb
Jacobaea analoga (DC.) Veldk.<br>Syn. *Senecio chrysanthemoides* DC.	Compositae	Herb
Juglans regia L.	Juglandaceae	Tree
Juniperus squamata D. Don	Cupressaceae	Shrub
Koeleria pyramidata (Lam.) P. Beauv.	Poaceae	Herb
Koenigia alpina (All.) Sch. & Rev.<br>Syn. *Polygonum alpinum* All.	Polygonaceae	Herb
Lamium album L.	Lamiaceae	Herb
Lamium amplexicaule L.	Lamiaceae	Herb
Lavatera cachemiriana (Camb.) Alef.	Malvaceae	Herb
Leontopodium jacotianum Beauv.	Compositae	Herb
Leucanthemum vulgare Lam.	Compositae	Herb
Linaria dalmatica (L.) Mill.	Plantaginaceae	Herb
Lonicera iberica M. Bieb.<br>Syn. *Lonicera alpigena* Gueld.	Caprifoliaceae	Shrub
Lonicera japonica Thunb.	Caprifoliaceae	Shrub
Lonicera obovata Royle ex. Hook.f. & Thompson	Caprifoliaceae	Shrub
Lotus corniculatus L.	Fabaceae	Herb
Malva neglecta Wall.	Malvaceae	Herb
Marrubium vulgare L.	Lamiaceae	Herb
Medicago lupulina L.	Fabaceae	Herb
Melanoseris macrorhiza (Royle) N. Kilian<br>Syn. *Lactuca macrorhiza* (Royle) Hook.f.	Compositae	Herb
Morus alba L.	Moraceae	Tree
Myosotis alpestris Schmidt	Boraginaceae	Herb
Myosotis stricta Link ex Roem. & Sch.	Boraginaceae	Herb
Nasturtium officinale Aiton	Brassicaceae	Herb
Nepeta cataria L.	Lamiaceae	Herb
Nepeta connata Royle ex Beath.	Lamiaceae	Herb
Nepeta laevigata (D. Don) Hand.-Mazz.	Lamiaceae	Herb
Nepeta linearis Royle ex Benth.	Lamiaceae	Herb
Origanum vulgare L.	Lamiaceae	Herb
Oxalis acetocella L.	Oxalidaceae	Herb
Oxalis corniculata L.	Oxalidaceae	Herb

Oxyria digyna (L.) Hill.	Polygonaceae	Herb
Parrotiopsis jacquemontiana (Decne.) Rehder	Hamamelidaceae	Shrub
Pedicularis punctata (Decne.) Rehder	Orobanchaceae	Herb
Pedicularis siphonantha D. Don	Orobanchaceae	Herb
Persicaria amphibia (L.) Delarbre	Polygonaceae	Herb
Phleum alpinum L.	Poaceae	Herb
Phlomoides bracteosa (Royle ex Benth.) Kam. & Makhm.<br>Syn. *Phlomis bracteosa* Royle ex Benth.	Lamiaceae	Herb
Phytolacca acinosa Roxb.	Phytolaccaceae	Herb
Picea smithiana (Wall.) Boiss	Pinaceae	Tree
Pinus wallichiana A. B. Jacks.	Pinaceae	Tree
Plantago himaliaca Pilg.	Plantaginaceae	Herb
Plantago lanceolata L.	Plantaginaceae	Herb
Plantago major L.	Plantaginaceae	Herb
Platanus orientalis L.	Platanaceae	Tree
Poa angustifolia L.	Poaceae	Herb
Poa pratensis L.	Poaceae	Herb
Podophyllum hexandrum Royle	Berberidaceae	Herb
Polemonium caeruleum L.	Polemoniaceae	Herb
Polygonum aviculare L.	Polygonaceae	Herb
Polystichum lonchitis (L.) Roth.	Dryopteridaceae	Herb
Primula elliptica Royle	Primulaceae	Herb
Primula macrophylla D. Don	Primulaceae	Herb
Primula rosea Royle	Primulaceae	Herb
Prunella vulgaris L.	Lamiaceae	Herb
Prunus cornuta (Wall. ex Royle) Steud.	Rosaceae	Herb
Quercus leucotrichophora Camus	Fagaceae	Tree
Quercus robur L.	Fagaceae	Tree
Rannunculus brotherusii Freyn	Ranunculaceae	Herb
Ranunculus trichophyllus Chaix	Ranunculaceae	Herb
Ranunculus arvensis L.	Ranunculaceae	Herb
Ranunculus hirtellus Royle	Ranunculaceae	Herb
Ranunculus repens L.	Ranunculaceae	Herb
Rheum webbianum Royle	Polygonaceae	Herb
Rhodiola himalensis (D. Don) Fu	Crassulaceae	Shrub
Rhododendron anthopogon D. Don	Ericaceae	Shrub
Rhododendron campanulatum D. Don	Ericaceae	Shrub
Ribes orientale Desf.	Grossulariaceae	Shrub
Robinia pseudoacacia L.	Fabaceae	Tree
Rorippa islandica (Oeder) Borbas	Brassicaceae	Herb
Rosa pouzinii Tratt.<br>Syn. *Rosa microphylla* Desf.	Rosaceae	Shrub
Rosa webbiana Wall. ex Royle	Rosaceae	Shrub
Rumex hastatus D. Don	Polygonaceae	Herb
Rumex nepalensis Spreng.	Polygonaceae	Herb
Salix alba L.	Salicaceae	Tree
Salix babylonica L.	Salicaceae	Tree
Salix denticulata Ander.	Salicaceae	Shrub
Salix flabellaris Ander.	Salicaceae	Shrub
Salvia glutinosa L.	Lamiaceae	Herb
Salvia hians Royle ex Benth.	Lamiaceae	Herb
Sambucus wightiana Wall. ex Wight & Arn.	Adoxaceae	Herb
Saussurea atkinsonii C.B. Clarke	Compositae	Herb
Saussurea candolleana DC.	Compositae	Herb
Sibbaldia cuneata Edgew.	Rosaceae	Herb

Sigesbeckia orientalis L.	Compositae	Herb
Silene baccifera (L.) Durande	Caryophyllacea	Herb
Silene coronaria (L.) Clairv.	Caryophyllacea	Herb
Skimmia anquetilia Taylor & Shah	Rutaceae	Shrub
Sonchus oleraceus L.	Compositae	Herb
Taraxacum sect. Taraxacum F. H. Wigg. Syn. *Taraxacum officinale* F. H. Wigg.	Compositae	Herb
Taxus wallichiana Zucc.	Taxaceae	Tree
Thalictrum alpinum L.	Ranunculaceae	Herb
Thymus linearis Benth.	Lamiaceae	Herb
Trifolium pratense L.	Fabaceae	Herb
Trifolium repens L.	Fabaceae	Herb
Trillium govanianum Wall. ex D. Don	Melanthiaceae	Herb
Tussilago farfara L.	Compositae	Herb
Ulmus wallichiana Planch.	Ulmaceae	Tree
Urtica dioica L.	Urticaceae	Herb
Valeriana jatamansi Jones ex Roxb.	Caprifoliaceae	Herb
Verbascum thapsus L.	Scrophulariaceae	Herb
Veronica laxa Benth.	Plantaginaceae	Herb
Viburnum cotinifolium D. Don	Adoxaceae	Shrub
Viburnum grandiflorum Wall. ex DC.	Adoxaceae	Shrub
Viola biflora L.	Violaceae	Herb
Viola odorata L.	Violaceae	Herb
