# Supplementary material for: Pattern of β-Diversity and Plant Species Richness along Vertical Gradient in Northwest Himalaya, India
Source: Biology (Basel). 2022 Jul 18;11(7):1064. doi: 10.3390/biology11071064 (PMC9312975; doi:10.3390/biology11071064)
Supplement: Supplementary file 1 [file biology-11-01064-s001.zip › Suppl S2.pdf]

Suppl. S2

Table S2. Family wise contribution of the documented plant species

Family	No. of species	Family	No. of species
Adoxaceae	3	Amaranthaceae	2
Apiaceae	2	Araceae	2
Araliaceae	1	Aspleniaceae	2
Athyriaceae	1	Balsaminaceae	3
Berberidaceae	4	Betulaceae	2
Boraginaceae	3	Brassicaceae	6
Campanulaceae	2	Cannabaceae	2
Caprifoliaceae	5	Caryophyllacea	3
Celestraceae	1	Compositae	28
Crassulaceae	1	Cupressaceae	1
Cyperaceae	1	Cystopteridaceae	1
Dryopteridaceae	2	Equisetaceae	1
Ericaceae	2	Euphorbiaceae	1
Fabaceae	10	Gentianaceae	1
Geraniaceae	3	Grossulariaceae	1
Hamamelidaceae	1	Hypericaceae	1
Iridaceae	3	Juglandaceae	1
Lamiaceae	17	Malvaceae	2
Melanthiaceae	1	Moraceae	1
Oleaceae	1	Onagraceae	1
Orobanchaceae	3	Oxalidaceae	2
Papaveraceae	3	Phytolaccaceae	1
Pinaceae	4	Plantaginaceae	8
Platanaceae	1	Poaceae	6
Polemoniaceae	1	Polygonaceae	10
Primulaceae	5	Pteridaceae	2
Ranunculaceae	11	Rosaceae	9
Rubiaceae	1	Rutaceae	1
Salicaceae	4	Sapindaceae	2

Saxifragaceae	2	Scrophulariaceae	1
Simaroubaceae	1	Solanaceae	2
Taxaceae	1	Ulmaceae	1
Urticaceae	1	Violaceae	2
